# Feasibility of Microbubble-Accelerated Low-Dose Thrombolysis of Peripheral Arterial Occlusions Using an Ultrasound Catheter

**DOI:** 10.1177/15266028221126938

**Published:** 2022-09-29

**Authors:** Sabrina A. N. Doelare, Johanna H. Nederhoed, Josje M. Evers, Sebastiaan T. Roos, Otto Kamp, René J. P. Musters, Willem Wisselink, Vincent Jongkind, Harm P. Ebben, Kak K. Yeung

**Affiliations:** 1 Department of Surgery, Amsterdam Cardiovascular Sciences, Amsterdam UMC – Vrije Universiteit Amsterdam, Amsterdam, The Netherlands; 2 Department of Physiology, Amsterdam Cardiovascular Sciences, Amsterdam UMC – Vrije Universiteit Amsterdam, Amsterdam, The Netherlands; 3 Department of Cardiology, Amsterdam Cardiovascular Sciences, Amsterdam UMC – Vrije Universiteit Amsterdam, Amsterdam, The Netherlands; 4 Department of Surgery, Dijklander Hospital, Hoorn, The Netherlands

**Keywords:** thrombolysis, endovascular treatment/therapy, microbubbles, ultrasound, peripheral artery disease, contrast-enhanced sonothrombolysis, drug delivery, in vitro, in vivo

## Abstract

**Purpose:**

Intra-arterial administration of microbubbles (MBs) through an ultrasound (US) catheter increases the local concentration of MBs into the thrombus and may further enhance outcomes of contrast-enhanced sonothrombolysis (CEST). The objective of this study was to evaluate the feasibility and lytic efficacy of intra-arterial infusion of MBs during US-enhanced thrombolysis in both in vitro and in vivo peripheral arterial occluded models.

**Materials and Methods:**

SonoVue and Luminity MBs were infused at a flow rate of 20 mL/h through either the drug delivery lumen of the US catheter (DDC, n=20) or through the tube lumen of the vascular phantom (systematic infusion, n=20) during thrombolysis with a low-dose urokinase (UK) protocol (50 000 IU/h) with(out) US application to assess MB survivability and size by pre-treatment and post-treatment measurements. A human thrombus was placed into a vascular phantom of the flow system to examine the lytic effects of CEST by post-treatment D-dimer concentrations measurements of 5 treatment conditions (saline, UK, UK+US, UK+US+SonoVue, and UK+US+Luminity). Thrombolytic efficacy of localized MBs and US delivery was then investigated in vivo in 5 porcine models by arterial blood flow, microcirculation, and postmortem determined thrombus weight and remaining length.

**Results:**

US exposure significantly decreased SonoVue (p=0.000) and Luminity (p=0.000) survivability by 37% and 62%, respectively. In vitro CEST treatment resulted in higher median D-dimer concentrations for the SonoVue (0.94 [0.07–7.59] mg/mL, p=0.025) and Luminity (0.83 [0.09–2.53] mg/mL, p=0.048) subgroups when compared with thrombolysis alone (0.36 [0.02–1.00] mg/mL). The lytic efficacy of CEST examined in the porcine model showed an improved median arterial blood flow of 21% (7%–79%), and a median thrombus weight and length of 1.02 (0.96–1.43) g and 2.25 (1.5–4.0) cm, respectively. One allergic reaction and 2 arrhythmias were observed due to the known allergic reaction on lipids in the porcine model.

**Conclusion:**

SonoVue and Luminity can be combined with an US catheter and could potentially accelerate thrombolytic treatment of peripheral arterial occlusions.

**Clinical Impact:**

Catheter-directed thrombolysis showed to be an effective alternative to surgery for acute peripheral arterial occlusions, but this technique is still associated with several limb and life-threatening complications. The effects of thrombolysis on clot dissolution may be further enhanced by intra-arterial administration of microbubbles through an ultrasound catheter. This study demonstrates the feasibility and lytic efficacy of intra-arterial infusion of microbubbles during US-enhanced thrombolysis in both in vitro and in vivo peripheral arterial occluded models.

## Introduction

Catheter-directed thrombolysis (CDT) has been an effective treatment for patients with nonthreatening acute limb ischemia for the last 2 decades.^1^ Limitations of this treatment include bleeding complications in up to 13% of patients and an extended time needed for revascularization, which places a heavy burden on the patient.^2,3^ Therefore, acceleration of thrombolytic treatment is needed to minimize risks and limit patient burden.

Recently, studies on the use of microbubbles (MBs) combined with ultrasound (US) have revealed that it could be a potential accelerator of thrombolytic treatment for peripheral arterial occlusions.^4–7^ When exposed to US, MBs start to oscillate (low-intensity US) and can eventually collapse (high-intensity US), causing erosion, and microjet formation, of the thrombus surface, which eventually weakens the fibrin network. This leads to an increase in thrombus susceptibility to thrombolytic agents and can, thereby, accelerate the effects of thrombolysis on clot dissolution.^8^

The influence of contrast-enhanced sonothrombolysis (CEST) has already been assessed in a porcine model of peripheral arterial occlusion and showed to significantly accelerate fibrinolysis when combined with a low-dose urokinase (UK) protocol.^9^ More recently, we have demonstrated the clinical safety and applicability of CEST in the MUST (Microbubbles and UltraSound-accelerated Thrombolysis for peripheral arterial occlusions) trial, including 20 patients with peripheral arterial occlusion.^10^ This novel technique at present has some limitations such as the need for external application of US, which exhibits an interoperator variability that may negatively affect the cavitation effect. Another limitation is that intravenous administration of MBs primarily induces cavitation on the surface of the thrombus and not directly into the thrombus until microchannels are formed or partial reperfusion is ensured. These limitations might be resolved by administration of the MBs directly into the thrombus through a drug delivery catheter that is positioned within the thrombus.

The efficacy of intra-arterial administered MBs as an add-on to an US drug delivery catheter has recently been proven in vitro as well as in vivo in an inferior vena cava thrombosis model treated with a custom-designed US catheter and lab-produced MBs.^11^ However, current data on the effects of clinically available US catheters on Food and Drug Administration/the European Medicines Agency (FDA/EMEA)-allowed MBs within peripheral arterial occlusion models are as yet unknown. Therefore, the aim of the present article is 2-fold: to assess the feasibility of intra-arterial administered MBs through an US catheter and to assess the lytic efficacy of MB US-accelerated thrombolysis in a validated porcine model of peripheral arterial occlusion.^12^

## Methods and Materials

To investigate the feasibility of MB infusion through an EkoSonic catheter during thrombolytic therapy, 3 main experiments were conducted. First, baseline feasibility experiments were performed in a vascular flow phantom to evaluate the impact of US, MB type, and infusion techniques on MB survival and size. Second, the effect of the aforementioned settings on clot lysis was examined by D-dimer measurements after treatment. Finally, the effectiveness of MB US-accelerated thrombolysis was evaluated in vivo in a porcine model of peripheral arterial occlusion.

### In Vitro Model

#### Therapeutic US catheter

**Figure 1.**
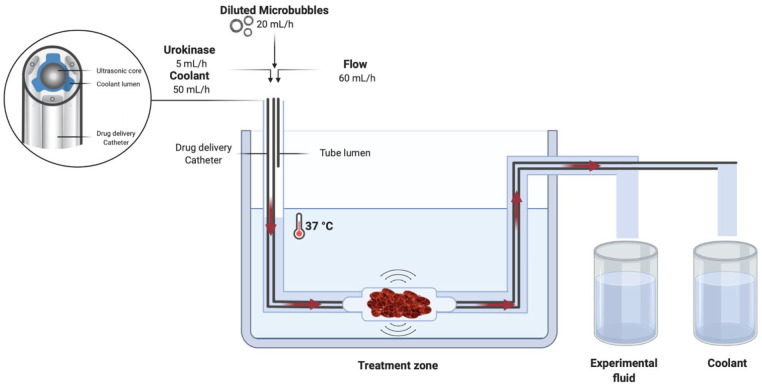
In vitro setup with ultrasound catheter in vascular phantom. Overall in vitro experimental setup. Solid red arrows indicate the direction of flow.

In the experiments, an intravascular US-assisted thrombolysis system (EKOS EkoSonic System; EKOS Corp, Bothell, WA, USA) was used, consisting of 2 components: Intelligent™ DDC and a removable US wire, MicroSonic™ Device. The DDC contains a series of micro pores located along a 6 cm treatment zone providing thrombolytic agent infusion, as shown in Figure 1. The removable US wire, located centrally in the lumen of the infusion catheter, consists of miniaturized US transducers that deliver low-intensity and high-frequency (2.2 MHz) US. According to the manufacturer’s instructions, saline was infused through the EKOS with a flow rate of 50 mL/h to prevent overheating.

#### MB preparation

The SonoVue (Bracco International B.V., Amsterdam, the Netherlands) and Luminity (Penn Pharmaceutical Services Ltd, Wales, UK) MBs, both clinically approved for diagnostic use in Europe, were prepared according to the manufacturer’s instructions and characterized as previously described.

#### Flow model

The overall in vitro experimental setup (Figure 1) consisted of a 37°C water bath and a circulation tube connected to a custom-designed vascular phantom. Three infusion pumps (Perfusor FM, B. Braun, The Netherlands) were inserted into the circulation tube. The first pump was attached either to the DDC or to the tube lumen and infused MBs at a rate of 20 mL/h. The second pump was attached to the catheter to infuse saline at a rate of 50 mL/h through the coolant lumen. The third pump infused saline into the tube lumen of the vascular phantom to mimic blood flow at a rate of 60 mL/h. All pumps were validated for exact flow rates to correct small deviations in flow. In preparation of the experiment, dead spaces in the in vitro setup were primed by running all pumps for 5 minutes. After priming, the pumps ran for 10 minutes per experiment at constant flow rates. Samples consisting of a mixture of MBs and saline were collected in a glass column, while the coolant was collected separately.

#### Bubble survivability and sizing

Survivability of the MBs was measured by comparing MB concentrations pre-infusion and post-infusion through the catheter system. For the pre-infusion measurement, a sample at *t*=0 minute (after priming) and a sample at *t*=10 minutes was tested and an average was used to correct for differences through time. The MB concentration post-infusion was measured in the fluid collected from the drug delivery lumen and the vascular phantom lumen and corrected for dilution. Microbubble solutions were analyzed for concentration and particle size using a MultiSizer 3 Coulter counter (Beckman Coulter Nederland B.V., The Netherlands) with a detection limit of 0.5 to 30.0 µm. Two differently sized apertures (30 and 50 µm) were used to validate the MB size and concentration measurements.

#### Experiment groups

As show in Table 1, the experiments were divided into 8 groups testing the influence of US and 2 infusion techniques with SonoVue and Luminity MBs (n=10 per group). The standard EkoSonic US protocol (variable amplitude pulsed protocol) was used for the continuously ON US setting. Microbubble infusion through the DDC of the US catheter simulated intra-arterial infusion, whereas MB infusion into the tube lumen of the vascular phantom simulated systemic intravenous MB infusion.

**Table 1.** Experiment Groups Used in the In Vitro Experiments (n=10 per Group).

Group	Ultrasound	Infusion techniques	Microbubble Infusion
1	OFF	Drug delivery lumen	SonoVue
2	OFF	Tube lumen	SonoVue
3	OFF	Drug delivery lumen	Luminity
4	OFF	Tube lumen	Luminity
5	ON	Drug delivery lumen	SonoVue
6	ON	Tube lumen	SonoVue
7	ON	Drug delivery lumen	Luminity
8	ON	Tube lumen	Luminity

### In Vitro Thrombus Model

#### Thrombus preparation

To initiate clotting, 15 mM CaCl_2_ and 1 international unit (IU) of human thrombin (Tissucol, Baxter, IL, USA) were dissolved in 5 mL of citrated venous human blood. The sample was transferred to a glass tube to incubate for 3 hours at 37°C in a stove (Heraeus Holding GmbH, Hanau, Germany). The formed artificial clot was transferred to the vascular phantom and the tip of the catheter was inserted into the thrombus (Figure 1). The MBs and a UK (Medacinase Urokinase; Medac, Hamburg, Germany) solution (1×10^4^ IU UK in 5 mL normal saline) were separately infused through the DDC with a flow rate of 5 mL/h.

#### D-dimer measurements

To examine the lytic effect of both MBs with UK treatment, D-dimer measurements were used as the metric for clot lysis after 1 hour of treatment. Five treatment conditions (n=12) were implemented for the basic in vitro thrombolysis tests: (1) phosphate buffer saline alone that served as a control group, (2) UK alone, (3) UK+US, (4) UK+US+SonoVue, and (5) UK+US+Luminity. D-dimer measurements were performed on the fluid collected from the drug delivery lumen and the vascular phantom lumen. The samples were centrifuged at 2400 RPM for 10 minutes and frozen at –20°C. Before D-dimer testing was done, the samples were thawed at room temperature for 15 to 30 minutes.

### Porcine Model of Peripheral Arterial Occlusion

The lytic efficacy was evaluated in a porcine model (n=5) of peripheral arterial occlusion. Approval of the Animal Ethics Committee (AEC) was obtained before initiation of the study (local registration number FYS 10-11). Five Yorkshire pigs with a median weight of 67 (64–74) kg were used. The porcine model of peripheral arterial occlusion was used as previously described (Lab Animal 2014). After creation and stabilization of a 4 cm thrombus in the external iliac artery, this experimental treatment group was treated for 3 hours with a US catheter (continuously ON) and 4 vials of SonoVue MBs infused through the DDC during the first hour. A low-dose thrombolysis protocol was used which consisted of 50 000 IU of UK (Medac) per hour. The measured determinants for lytic effect were blood flow through the vessel (mL/min), microcirculation (perfusion Units; PU), blood pressure (mm Hg), temperature of affected and contralateral limbs (°C), thrombus weight postmortem (grams), and any adverse events. To limit the animal burden, the current results were compared with a historical control group solely treated with UK in our previous study.

### Statistics

Data were analyzed using SPSS (IBM Statistics v20, Chicago, IL, USA) and presented as medians (range) in cases of nonparametric distribution or as means±SD in cases of parametric distribution. An independent *t* test or a Mann–Whitney *U* test was used to compare continuous variables between groups. A Bonferroni correction was used in case of multiple comparisons to decrease the likelihood of a type I error. A p value less than 0.05 was considered statistically significant.

## Results

### In Vitro Model

#### US setting: OFF vs ON

**Figure 2.**
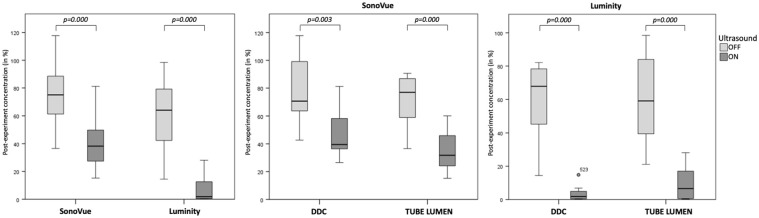
Microbubble survivability after infusion in a vascular phantom. Microbubble survivability is measured by the microbubble concentration ratio post-infusion/pre-infusion. The post-infusion concentration was corrected for dilution by the tube flow following from the infusion rates and collected volumes. Left boxplot shows survivability of SonoVue and Luminity microbubbles per ultrasound mode (n=20 per group), and middle and right boxplots show survivability of SonoVue (n=10 per group) or Luminity microbubbles (n=10 per group) per ultrasound mode infused through the drug delivery lumen of the catheter (DDC, simulating intra-arterial infusion) or directly into the vascular phantom (simulating intravenous infusion). DDC, drug delivery lumen catheter.

**Figure 3.**
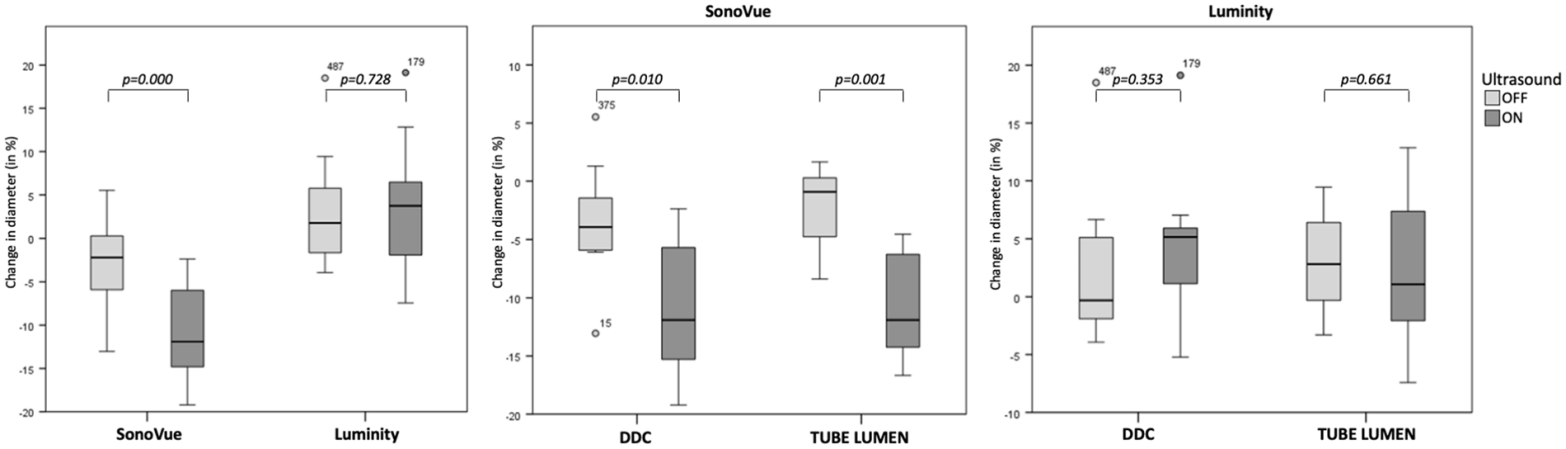
Change in microbubble size after infusion in a vascular phantom. Change in microbubble size is measured by the microbubble concentration ratio post-infusion/pre-infusion. The change in size was corrected for dilution by the tube flow following from the infusion rates and collected volumes. Left boxplots show change in size of SonoVue and Luminity microbubbles per ultrasound mode (n=20 per group), and middle and right boxplots show change in size of SonoVue (n=10 per group) and Luminity microbubbles (n=10 per group) per ultrasound mode infused through the drug delivery lumen of the catheter (DDC, simulating intra-arterial infusion) or directly into the vascular phantom (simulating intravenous infusion). DDC, drug delivery lumen catheter.

Table 2 shows the median change in MB concentration and diameter infused through DDC or tube lumen as determined by precision apertures with a diameter of 30 and 50 µm. The effect of US influenced the MB survivability and size significantly in both DDC and tube lumen groups. Figure 2 illustrates a relative decrease in overall SonoVue and Luminity concentration of 75% to 38% (p=0.000) and 64% to 2% (p=0.000) when exposed to US, respectively. In addition, overall median diameter of SonoVue and Luminity MBs changed from –2.2% to –11.9% (p=0.000) and 1.8% to 3.7% (p=0.728) when exposed to US (Figure 3).

**Table 2.** Change in Median Size Diameter and Number Concentration Values of Ultrasound-Exposed and Non-Exposed SonoVue and Luminity Solutions Infused Either Through the Drug Delivery Lumen of the Catheter (DDC, Simulating Intra-Arterial Infusion) or Directly Into the Vascular Phantom (Simulating Intravenous Infusion) as Determined by 30 and 50 µm Sized Apertures.

Aperture size	Catheter	Microbubble	US OFF	US ON	p-value	US OFF	US ON	p value
			∆%^a^ median MB diameter (in µm)	∆%^a^ median MB diameter (in µm)		∆%^a^ median MB concentration (×10^7^/mL)	∆%^a^ median MB concentration (×10^7^/mL)	
30 µm	DDC	SonoVue	−5.9 (−13.0 to −1.45)	−15.3 (−19.2 to −14.0)^b^	0.009	64 (43–71)	37 (27–40)^c^	0.009
		Luminity	−1.4 (−3.9 to 1.8)	5.4 (2.2 to 19.1)	0.009	77 (56–82)	1 (0–1)	0.009
	Tube lumen	SonoVue	0.3 (−8.4 to 1.7)	−12.2 (−14.4 to −11.7)^b^	0.009	59 (37–88)	24 (15–29)^c^	0.009
		Luminity	−0.4 (−3.3 to 2.6)	−2.0 (−7.4 to 7.4)	0.624	77 (28–95)	22 (0–76)	0.009
50 µm	DDC	SonoVue	−0.8 (−3.93 to 5.53)	−5.7 (−2.4 to −9.8)	0.050	99 (70–117)	58 (36–81)	0.016
		Luminity	5.1 (−3.0 to 18.5)	1.1 (−5.2 to 7.0)	0.465	45 (15–75)	5 (3–15)	0.016
	Tube lumen	SonoVue	−1.2 (−4.8 to −0.3)	−6.3 (−16.7 to −4.6)	0.027	81 (75–91)	46 (35–60)	0.009
		Luminity	6.4 (2.8 to 9.5)	5.4 (−1.7 to 12.9)	0.917	57 (21–77)	16 (12–28)	0.016

Non-normal variables are presented as median (interquartile range).

Abbreviations: DDC, drug delivery lumen catheter; MB, microbubble; US, ultrasound.

a ∆%=relative difference post-infusion vs pre-infusion expressed in percentage.

b US ON DDC ON 30 µm vs US ON tube lumen ON 30 µm, p=0.028.

c US ON DDC ON 30 µm vs US ON tube lumen ON 30 µm, p=0.016.

#### Mode of infusion: DDC vs tube lumen

Figures 2 and 3 show bar chart representations of the median change in microbubble concentration and size using DDC and tube lumen when subjected to continuous US exposure or none. Statistical evaluation of the analyses showed no overall differences in the survivability and size of SonoVue and Luminity MBs between infusion through DDC and tube lumen. However, when exposed to US, the mode of infusion significantly influenced the survivability and size of SonoVue MBs as measured by 30 µm size aperture (Table 2).

#### D-dimer measurements

**Figure 4.**
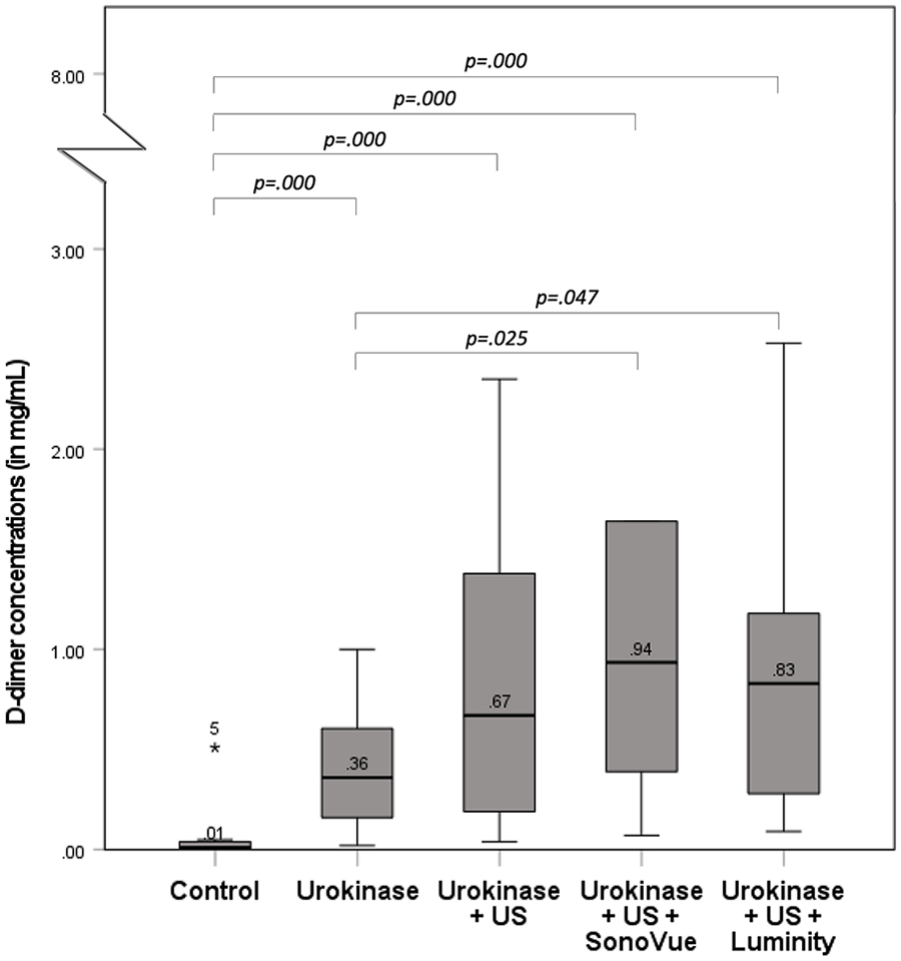
D-dimer concentrations after various treatment regimens in a vascular phantom. Clot lysis is measured by the post-D-dimer concentration for all treatment groups, including saline (n=13), urokinase (n=15), urokinase+US (n=12), urokinase+US+SonoVue (n=12) and urokinase+US+Luminity (n=12) infused through the drug delivery lumen of the catheter (DDC, simulating intra-arterial infusion). US, ultrasound; DDC, drug delivery lumen catheter.

Figure 4 depicts the D-dimer concentrations measured for the treatment groups categorized for the treatment regimens. According to these results, D-dimer levels differed significantly among the treatment groups (p=0.000). Treatment containing UK, US, and MB (SonoVue, 0.94 [0.07–7.59] mg/mL; Luminity, 0.83 [0.09–2.53] mg/mL) resulted in higher D-dimer levels when compared with treatment with UK alone (0.36 [0.02–1.00] mg/mL).

### In Vivo Model

As described in the “Methods and Materials” section, the lytic efficacy was evaluated in a series of different experiments in a porcine model. Despite treatment with a preventive protocol for allergic reactions, 1 of 5 pigs unfortunately died before the start of the experiment due to cardiac and respiratory failure, probably caused by hypersensitivity to the anesthesia. In the remaining 4 pigs, a complete occlusion at initiation of experimental therapy was reached without residual flow through the iliac artery. As can been seen in Table 3, all 4 pigs showed an increase in arterial flow with a median increase of 21% (range, 7%–79%), of which 1 showed a slight improvement of arterial flow (7 mL/min). The median microcirculatory flow of the whole group was 46 (range, 36–173) PU at baseline and decreased to 34 (range 17–46) PU after stabilization of the thrombus. Two out of 4 pigs showed a median increase of 165% in microcirculation (range, 17%–160%), while the other 2 showed a median decrease of 60%. During the procedure, the mean systemic arterial pressures of all but one pig rose with a median rate of 3% (range –5% to 2%). At the end of the procedure, the entire group showed a slight increase of 1°C in the systemic temperature. The opposite was observed for median temperatures of the affected (–3.2°C) and control limbs (–0.3°C). The median thrombus weight at the end of the experiment was 1.02 (0.96–1.43) g, but a markedly higher median thrombus weight of 1.43 g was observed in 1 pig. Besides the allergic reaction, we encountered 2 arrhythmias during the experiments, which led to premature discontinuation of the experiment after 165 minutes in 1 pig. As 1 dropout was accounted for in our AEC protocol, the experiment could not be repeated.

**Table 3.** Thrombus Induction in the Limb Due to Therapy With SonoVue Microbubbles Infused Through an EkoSonic Endowave Catheter in 4 Individual Pigs.

Parameters	Urokinase+microbubbles+EKOS				
	A1	A2	A3	A4	Median
Thrombus induction duration (min)	190	98	93		98
Amounts of thrombin (U)	250	100	100		100
Changes in flow (mL/min)	7	60	32	24	28
%B^a^	7	79	21	20	20.5
Microcirculation (PU)	160	17	−36	−8	4.5
%B^a^	320	10	−100	−19	−4.6
MAP limb (mm Hg)	2	1	−5		1
%B^a^	5	3	−12		3
T systemic (°C)	0.8	1.6	0.8		0.8
%B^a^	2.2	4.3	2.1		2.2
T affected limb (°C)	−2.2	−3.2	−5.2		−3.2
%B^a^	−12.4	−10	−15		−12.4
T control limb (°C)	−0.3	−0.9	−0.2		−0.3
%B^a^	−0.9	−3	−1		−1
Thrombus weight (g)	1.08	1.43	0.97	0.96	1.02
Thrombus length (cm)	2.5	4	2	1.5	2.25

Change in various parameters due to therapy in individual pigs, that is, *t*=180 minutes vs value after stabilization thrombus. Non-normal variables are presented as median (interquartile range).

Abbreviations: MAP, mean arterial pressure; PU, perfusion units; T, temperature.

a %B (baseline) is defined as the following ratio: change in flow/baseline flow×100%.

## Discussion

Catheter-directed thrombolysis showed to be an effective alternative to surgery for acute peripheral arterial occlusions. Even though altering to an in-thrombus approach has already contributed to improved patient outcomes, this technique is still associated with several limb and life-threatening complications.^13,14^ The potential use of MBs combined with US and thrombolytic therapy simultaneously shows to be promising in previous (pre-)clinical studies.^9,10,15^ The efficacy of thrombolysis might, however, be further improved by intra-arterial administration of MBs through an endovascular US catheter. Higher concentrations of MBs within the clot may enlarge the cavitation response, which will ultimately accelerate thrombolysis.

Gao et al^11^ also reported a significant increase in the efficacy of in vitro and in vivo thrombolysis after intra-clot MB-mediated sonothrombolysis with a US catheter. Despite the significant lytic effects observed, the study was limited by the use of a custom-designed US device. Before considering clinical application, this device should be subjected to the highest forms of regulatory inspection and may therefore hamper its wide clinical use.

In this article, we determined that application of SonoVue and Luminity MBs infused through an endovascular US catheter during low-dose thrombolytic treatment is feasible and effective. The application of MBs resulted in a significant decrease in MB concentration in both the DDC and tube lumen groups. This drop in MB concentration with the application of US suggests a potential therapeutic effect as the MBs were most likely destroyed at the site of interest, that is, after infusion into the thrombus near the catheter’s surface. Furthermore, results from the present in vitro study with thrombi clearly indicated a significant increase of thrombolysis after treatment with UK+US+MB when compared with conventional treatment with UK alone.

While it was expected that the application of US would significantly decrease MB concentrations, we also observed a significant decrease in the median size of MBs. This result is consistent with theoretical predictions, in which US is assumed to affect the larger MBs to a greater degree than the smaller one. More specifically, MBs exposed to high acoustic pressures may not only result in complete destruction of the MBs but also in fragmentation into smaller more stable MBs.^16,17^ However, one must take into account that the MBs size may change due to various reasons depending on the local milieu.^18^ Moreover, MB destruction may be accompanied by the production of debris particles out of the MB shell. As the size distributions of MB shell debris and small MBs are comparable, there is a concern of inaccurate particle sizing by the MultiSizer 3 Coulter counter.^19^ This may, nonetheless, not have any clinical implications as a significant increase in D-dimers was demonstrated with both MBs, indicating that fibrinolysis was effectuated to a greater extent than in treatment with UK alone.

The magnitude of clot lysis by intra-clot MB mediated thrombolysis with a US (UK+US+MB group) was also tested in vivo. In comparison to our previously performed experiments in porcine models of peripheral arterial occlusion (n=4) treated with conventional low-dose CDT (UK group), the results of this study show promising results.^9^ Three hours of thrombolytic therapy with intra-arterial infusion of MBs resulted in a lower thrombus weight (1.02 [0.96–1.43] g vs 1.59 [1.27–1.90] g) and shorter thrombus length (2.3 [1.5-4.0] cm vs 4.0 [2.5–4.0] cm) when compared with the conventional UK group of the previous study.^9^ We also observed an increase in arterial blood flow in all pigs of the UK+US+MB group, whereas a minimal increase in arterial blood flow was seen in only 1 of the 4 pigs in the UK group. Interestingly, total occlusion was reached in the remaining 3 pigs, but showed no improvement in arterial blood flow during the low-dose UK experiment.^9^ This is possibly influenced by the short duration of therapy and low-dose protocol, as more time would be required to achieve thrombolysis and regain vascularization. Nonetheless, marked improvement of reperfusion, that is, arterial blood flow, microcirculation, and limb arterial pressures, was observed in the UK+US+MB group. The results of the current study suggest that intra-clot MB-mediated sonothrombolysis with a US catheter could potentially accelerate thrombolytic treatment of peripheral arterial occlusions.

The current study has several limitations. Although inclusion of a human clot increases clinical relevance of the vascular flow model, it might not perfectly represent in vivo physiologic conditions. The used flow medium does not resemble whole blood, which may have influenced the MB behavior and cavitation response. To broaden clinical application, the bio-effects of MBs during low-dose thrombolysis of peripheral arterial occlusions using an US catheter were investigated in-vivo. The lytic efficacy in our porcine model was evaluated after 3 hours of treatment-time, which is shorter than the clinical situation of patients with peripheral arterial occlusions who are commonly treated for 2 to 3 days.^1^ However, longer treatment durations in a porcine model was not justified for logistic and ethical reasons. Despite the short treatment duration, the study shows promising reperfusion outcomes when compared to the control group receiving conventional catheter-directed thrombolysis.^9^

Regardless of the limitations, our study indicates that MB infusion combined with an US catheter shows significant decrease in MB survivability, but sufficient MBs survived to accelerate thrombolysis of peripheral arterial occlusions. Further studies are warranted to fully investigate the biomechanical properties of this therapy before it can be incorporated in the thrombolytic treatment for patients.

In conclusion, it is feasible to combine microbubbles with an US catheter in vitro. This technique is effective and might accelerate thrombolytic treatment of peripheral arterial occlusions in an in vivo model. Further clinical studies are needed to investigate the potential beneficial effects of MB and US-accelerated thrombolysis on the outcomes of patients with peripheral arterial occlusions.
